# The perspectives of biomedical application of the nanoceria

**DOI:** 10.1186/1878-5085-5-S1-A136

**Published:** 2014-02-11

**Authors:** Nadiya M Zholobak, Olexandr B Sherbakov, Lidia P Babenko, Olena S Bogorad-Kobelska, Rostyslav V Bubnov, Mykola Ya Spivak, Vladimir K Ivanov

**Affiliations:** 1 D.K. Zabolotny Institute of microbiology and virology NAS of Ukraine, Kyiv, Ukraine; 2 Clinical hospital “Pheophania” of State Affairs Department, Kyiv, Ukraine; 3 Kurnakov institute of general and inorganic Chemistry of Russian Academy of Sciences, Moscow, Russia

## Introduction

The scientific information on biological activity of ceria dioxide nanoparticles (CeO_2_, nanoceria) is still very fragmentary. It is known, that nanoceria interaction with biological systems is based on two principal properties of this substance: low toxicity and high reducibility [1]. These factors determines activity of nanoceria in biological redox processes, especially in inactivation of reactive oxygen species, including free radicals that are formed inside living cells.

## The aim

of the study was to assess the perspectives of nanoceria for various biomedical applications.

## Methods

We have performed series of studies to investigate the biomedical effects of nanoceria in vitro and in vivo. Effectivity of ceria nanoparticles after UV-irradiation was determined by the analysis of L929 and VERO cells Viability State of the cells were visualized under UV microscope using fluorescent Hoechst and Propidium iodide dyes. Investigated the effects on reproduction nanoceria VSV (RNA-) and HSV-1 (DNA-) viruses, the viability *of E.coli*, *S.aureus* and *C.albicans*. We studied the effects of nanoceria doxorubicin-induced ultrasonography guided cardiomyopathy model and on the reproductive system of older animals.

## Results

We estimated that 1, 25 mM colloid ceria solutions are capable to protect L929 and VERO cells from the damage induced by UV-irradiation [1,2]. Nanoceria could be used not only as UV-blocking material, but also as prophylactic and even therapeutic compound for treatment sunburns. We consider that nanoceria inhibits the reproduction of the studied RNA-and DNA-viruses in and demonstrates a significant virucidal effect [3], reducing the virus titer in the investigated model systems. We have shown the antibacterial activity for gram-positive microorganisms and expressive antifungal action [4]. Course reception of nanoceria by older laboratory animals (mice / rats) causes improvement in the viability of germ cells in males increases the level of testosterone in the blood, protect ovarian cells against oxidative damage [5], demonstrating anti-aging properties. Application of nanoceria against doxorubicin-induced cardiomyopathy on reliable model [6] can remove the drug-induced oxidative stress, restoring the broken functionality of the body.

## Conclusion

The application of nanoceria under conditions of involving oxidative stress can reduce / remove its damaging effects, thus providing protecting the organism from adverse environmental factors: UV irradiation, viral, bacterial, fungal lesions induced toxic effects and pathological conditions associated with aging.

## Outlook and expert recommendations

The biomedical effects of nanoceria offer the prospect of its use as a UV protectant, a drug with antiviral, antibacterial and antifungal activity, as well as means capable of reducing the level of oxidative stress in cardiomyocytes in reproductive organs during aging.
